# Image quality and quantification accuracy dependence on patient body mass in ^89^Zr PET/CT imaging

**DOI:** 10.1186/s40658-021-00420-4

**Published:** 2021-10-30

**Authors:** Ukihide Tateishi, Hiromitsu Daisaki, Junichi Tsuchiya, Yuji Kojima, Keisuke Takino, Naoki Shimada, Kota Yokoyama

**Affiliations:** 1 Department of Diagnostic Radiology, Graduate School of Medicine, Tokyo Medical and Dental University, Tokyo, Japan; 2 Department of Radiological Technology, Gunma Prefectural College of Health Sciences, Gunma, Japan; 3 Department of Diagnostic Radiology, Cancer Institute Hospital, Tokyo, Japan

**Keywords:** ^89^Zr, PET/CT, Patient’s body mass, Time-of-flight, Semi-conductor

## Abstract

**Background:**

This study was conducted to clarify how patient body mass affects the image quality and quantification accuracy of images obtained using ^89^Zr PET/CT. ^89^Zr PET/CT images from time-of-flight (TOF) PET/CT and semiconductor (SC) PET/CT were obtained using three types (M, L, LL; corresponding to increasing patient body weight) of custom-made body phantoms designed similarly to the National Electrical Manufacturers Association (NEMA) IEC body phantom. The phantom data were analyzed visually and quantitatively to derive image quality metrics, namely detectability of the 10-mm-diameter hot sphere, percent contrast for the 10-mm-diameter hot sphere (Q_H,10 mm_), percent background variability (N_10mm_), contrast-to-noise ratio (Q_H,10 mm_/N_10mm_), and coefficient of variation of the background area (CV_BG_).

**Results:**

Visual assessment revealed that all the 10-mm-diameter hot spheres of the three types of phantoms were identifiable on both SC and TOF PET/CT images. The N_10mm_ and CV_BG_ values were within the proposed reference levels, and decreased with acquisition duration for both PET/CT types. At 10-min acquisition, the Q_H,10 mm_/N_10mm_ of SC PET/CT was greater than the proposed reference level in all phantoms. However, the Q_H,10 mm_/N_10mm_ of TOF PET/CT was greater than the proposed reference level in M-type phantom alone. All the SUV_BG_ values were within 1.00 ± 0.05 for both PET/CT types.

**Conclusions:**

This study showed that the image quality and quantification accuracy depend on the patient’s body mass, suggesting that acquisition time on ^89^Zr PET/CT should be changed according to the patient’s body mass.

## Background

The positron radionucleotide ^89^Zr has been utilized for PET imaging labeled monoclonal antibodies to date, because long half-life radioisotope is needed for assessment of the circulating probes. ^89^Zr facilitates high-resolved PET imaging with a short positron range by emitting low-energy β^+^ rays. After introduction of desferrioxamine B (DFO) as specific chelator for ^89^Zr labeling, the number of antibodies and antibody fragments have been approved by the Food and Drug Administration (FDA) for last decade. Over the 17 antibodies including trastuzumab, bevacizumab, cetuximab, and rituximab were labeled with ^89^Zr for PET imaging [1–5]. Evaluating the target status of tumors is crucial for clinical decision making for patients planning molecular targeting therapy. A whole-body evaluation of target expression with ^89^Zr-trastuzumab PET changes the treatment plan [6]. Based on the results from recent two publications, the authors demonstrated that 89Zr-trastuzumab reflects tumor heterogeneity and supports clinical decision making when HER2 status could not be determined by standard procedures, which allows the selection of a personalized therapy [7, 8]. Thus, ^89^Zr-monoclonal antibody PET is promising for evaluating patient selection and therapeutic effect.

^89^Zr-monoclonal antibody PET would be utilized for clinical trials of multicenter in the near future. Thus, standardization and harmonization of ^89^Zr PET/CT have been investigated to date. According to the EANM procedure guideline for tumor imaging: version 2.0, EARL accreditation enhances the quality standards of PET/CT investigations to minimize the technical factors and ensures performance of PET/CT machines by harmonization [9]. EARL has also ^89^Zr PET/CT accreditation to ensure quantitative image quality using ^89^Zr labeled pharmaceuticals. In this context, several investigators have addressed multicenter harmonization of 89Zr PET/CT to ensure image quality and quantitation accuracy [10–13]. However, the results of the previous studies did not contain the problems affected by the patient’s body mass. PET imaging of larger patients is affected by high noise levels, because of the considerable intrinsic attenuation.

The purpose of this study was to clarify how patient body mass affects the image quality and quantification accuracy of images obtained using ^89^Zr PET/CT.

## Methods

### Phantom experiments

**Fig. 1:**
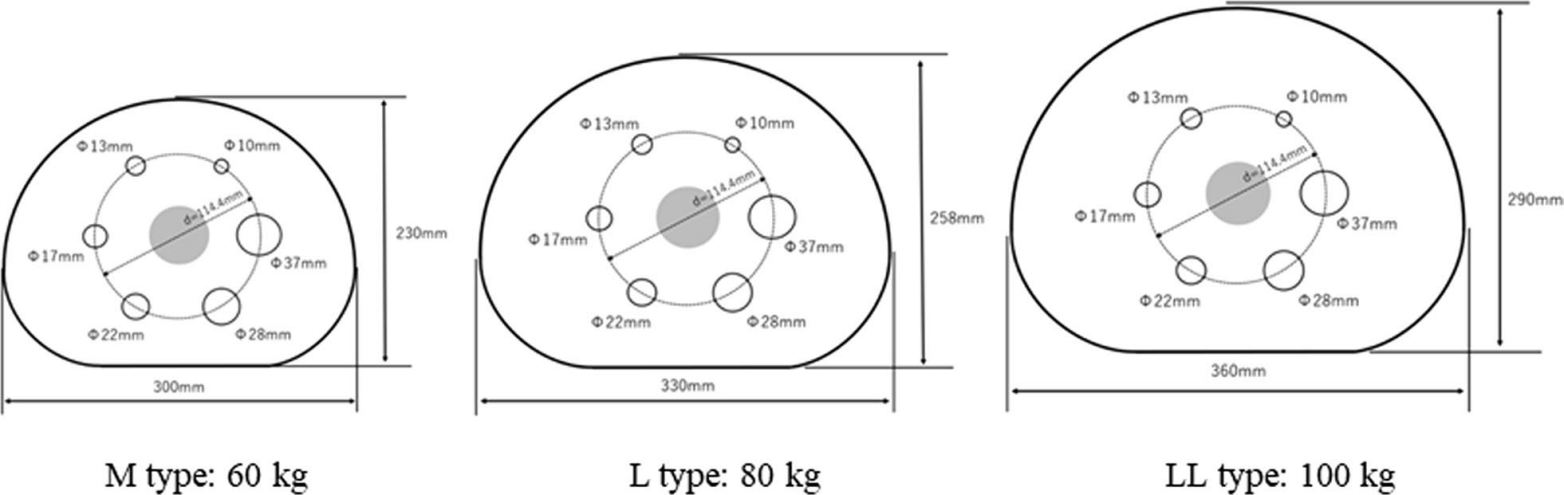
Custom-made body phantom simulating various body weight

Two PET/CT machines (Celesteion, time-of-flight [TOF] PET/CT and Cartesion Prime, semi-conductor [SC] PET/CT, Canon Medical Systems, Otawara, Tochigi, Japan) were investigated for study. The SC PET/CT is equipped with a silicon photomultiplier (SiPM). Three types of body phantoms including 30 (M type), 33 (L type), and 36 (LL type) cm in the major axis, corresponding to 60 kg, 80 kg, and 100 kg body weight, respectively, were designed similarly to National Electrical Manufacturers Association (NEMA) IEC body phantom by custom made (Fig. 1). These phantoms contained six spheres with inner diameters of 10, 13, 17, 22, 28, and 37 mm. All spheres were filled with ^89^Zr solutions to achieve 10:1 sphere-to-background activity concentration ratio based on the prior study of international standardization [8]. This study did not include human data and personal information.

### Data acquisition

**Fig. 2:**
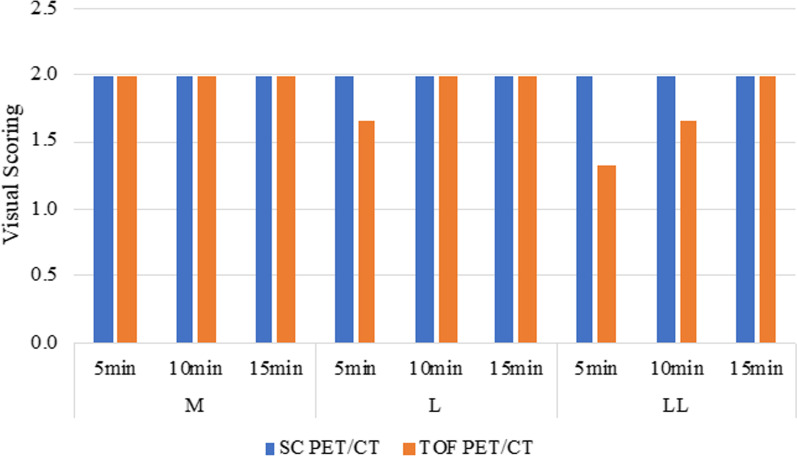
Visual scoring comparison between SC PET/CT and TOF PET/CT

The phantoms underwent a low-dose CT acquisition with auto-exposure of an X-ray tube current followed by PET acquisition for each scan. A 20-min-per-bed-position 1-bed-position for list mode acquisition and 5-, 10-, and 15-min-per-bed-position 2-bed-position acquisition were subsequently obtained for three types of phantoms (Fig. 2). PET images of SC PET/CT were reconstructed using parameters featuring ordered subset expectation maximization (OSEM) into a 336 × 336 matrix; voxel size 9.44 μl (2.11 × 2.11 × 2.11 mm) with two iterations, 12 subsets, a 3.0-mm 3D Gaussian filter, and active corrections by CT-based attenuation, scatter, TOF, and point-spread function (PSF). PET images of TOF PET/CT were also reconstructed using parameters featuring OSEM into a 336 × 336 matrix; voxel size 8.47 μl (2.04 × 2.04 × 2.04 mm) with three iterations, 10 subsets, a 6.0-mm 3D Gaussian filter, and corrections by CT-based attenuation, scatter, TOF, and PSF. Reconstructed images were evaluated by quantitative methods. Image analysis was performed using RAVAT (Nihon Medi-Physics Co., Ltd.) [14].

### Image analysis

Detectability of the 10-mm-diameter hot sphere was visually assessed by three nuclear medicine technologists in a three-step scale (0, not visualized; 1, visualized, but similar hot spots are observed; and 2, identifiable). The VOX-BASE/MANAGER (J-MAC SYSTEM, INC., Japan) was used to display PET images using an invert gray scale with an upper level of 10 and a lower level of 0 (SUV-scaled).

Quantitative analysis of image quality was performed for each image in accordance with the guidelines of the Japanese Society of Nuclear Medicine (JSNM) [15]. The percent contrast for the 10-mm hot sphere (Q_H,10 mm_), the percent background variability (N_10mm_) for the 10-mm circular region-of-interest (ROI), Q_H,10 mm_/N_10mm_ ratio, and the coefficient of variation on the background area (CV_BG_) (image noise level) were calculated. The background standardized uptake value (SUV_BG_), which reflects the accuracy of the calibration, was evaluated by the average value of SUV calculated by 12 ROIs with a diameter of 37 mm placed in the BG region, also in accordance with the guidelines of the JSNM [15]. The recovery coefficient (RC) of all hot spheres was quantified using RC_max_ and RC_peak_ (according to the QIBA calculation algorithm) as indicators as shown in Eq. 1 and Eq. 2 [14, 15]. Based on EARL accreditation manual Ver 2.0, the calibration quality control for ^89^Zr is similar to the ^18^F calibration phantom procedure, because ^89^Zr RCs are directly related to the RCs obtained with ^18^F [10, 12].

1 $$RC_{max,i} = \frac{{SUV_{max,i} }}{10}$$

2 $$RC_{peak,i} = \frac{{SUV_{peak,i} }}{10}$$

### Statistical analysis

Differences between groups for quantification data were analyzed using the parametric Student t test. A P value < 0.05 was considered significant. Statistical analysis was performed using SPSS version 28.0 (IBM-SPSS Japan Inc, Tokyo, Japan).

## Results

### Detectability

**Fig. 3:**
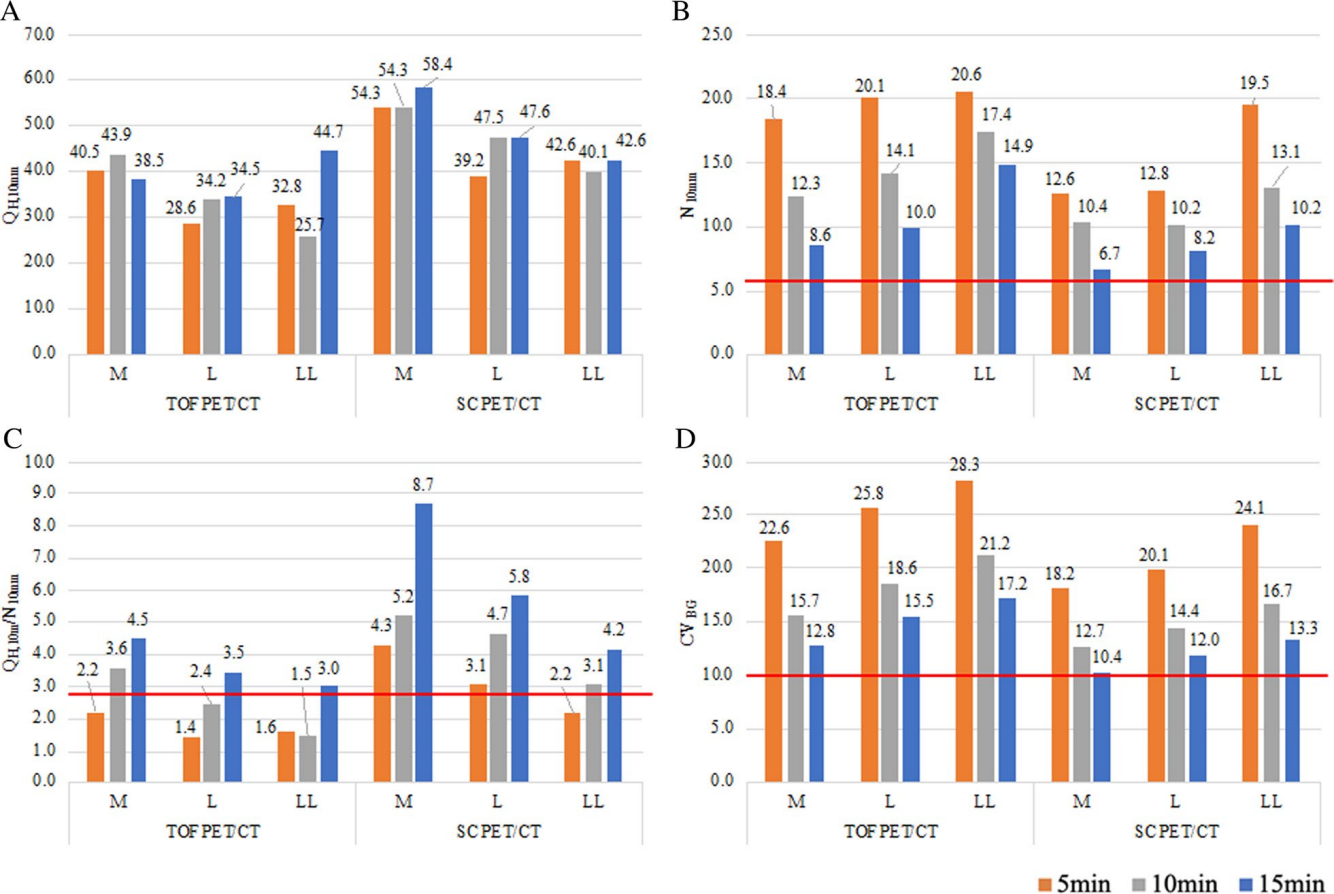
Comparison of image quality between SC PET/CT and TOF PET/CT. The Q_H,10 mm_ (**a**), N_10mm_ (**b**), Q_H,10 mm_/N_10mm_ (**c**), and CV_BG_ (**d**) of both PET/CT are presented, respectively. The proposed reference levels were for N_10mm_ (< 5.8), Q_H,10 mm_/N_10mm_ (> 2.8), and CV_BG_ (< 10.0), respectively

Visual assessment by SC PET/CT showed that all the 10-mm-diameter hot sphere were identifiable (Fig. 2). In contrast, detectability of the 10-mm-diameter hot sphere depended on scan duration in L type and LL type phantoms on TOF PET/CT. All the 10-mm-diameter hot sphere of three types of phantoms were visually identifiable on both PET/CT. The Q_H,10 mm_, N_10mm_, Q_H,10 mm_/N_10mm_, and CV_BG_ as a function of scan duration in all phantoms are shown in Fig. 3. The Q_H,10 mm_ did not correlate with acquisition duration. For N_10mm_, significant differences were observed in the 10-mm-sphere-detectable values among the three types of phantoms. The N_10mm_ and CV_BG_ decreased with acquisition duration for both PET/CT. The Q_H,10 mm_/N_10mm_ increased mostly with acquisition duration. SC PET/CT was capable of showing enough Q_H,10 mm_/N_10mm_ greater than 2.8 except for LL type phantom with 5-min acquisition. However, the Q_H,10 mm_/N_10mm_ of TOF PET/CT at 5-min acquisition was less than 2.8 in all phantoms. At 10-min acquisition, the Q_H,10 mm_/N_10mm_ of SC PET/CT was greater than 2.8 in all phantoms, whereas the Q_H,10 mm_/N_10mm_ of TOF PET/CT was greater than 2.8 in M type phantom alone. All Q_H,10 mm_/N_10mm_ at 15-min acquisition were greater than 2.8 in all phantoms for both of SC PET/CT and TOF-PET/CT.

### Accuracy

**Fig. 4:**
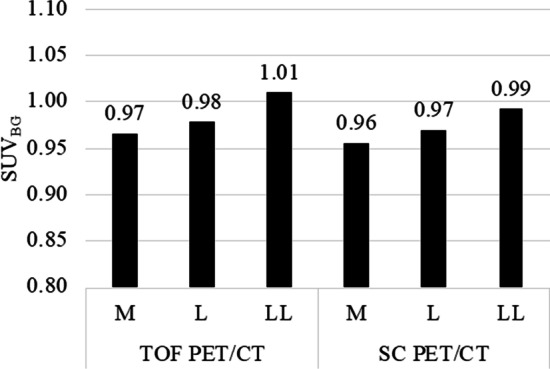
SUV_BG_ of both PET/CT scanners in three types of phantoms. The SUV_BG_ increases according to phantom size. All the SUV_BG_ were within 1.00 ± 0.05 for both PET/CT

**Fig. 5:**
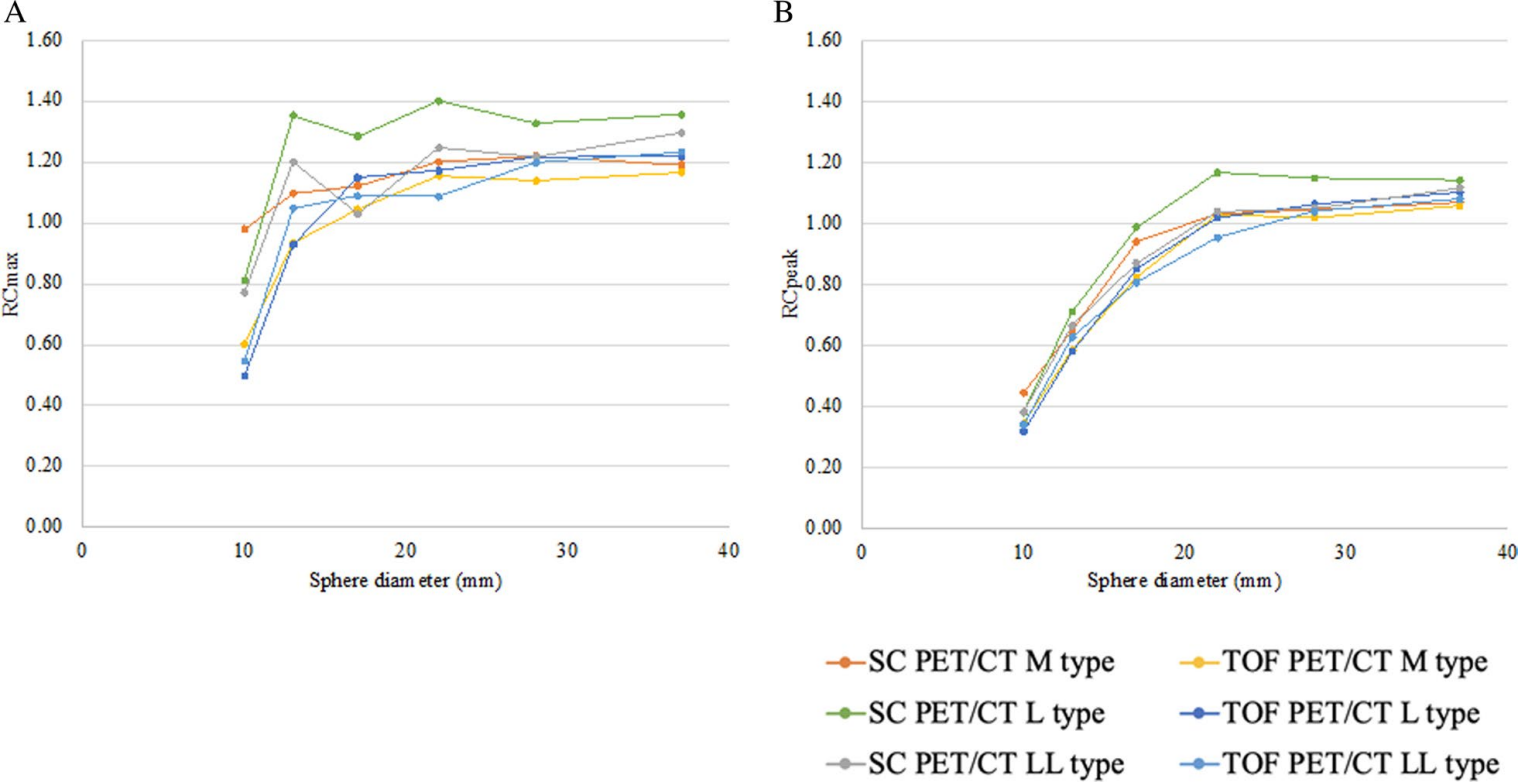
RC of both PET/CT scanners in three types of phantoms. The RC_max_ shows variations in sphere diameters of 10, 13, 17, and 22 mm (**a**), while the RC_peak_ represents minimal variation (**b**)

The SUV_BG_ of both scanners in three types of phantoms is shown in Fig. 4. The SUV_BG_ tended to be greater depending on phantom size. All the SUV_BG_ were within 1.00 ± 0.05 for both PET/CT. For TOF PET/CT, mean SUV of LL type was significantly greater than those of M type (p = 0.0085) or L type (p = 0.0092). Meanwhile, for SC PET/CT, mean SUV of LL type was significantly greater than those of M type (p = 0.0038). However, no significant difference was found in mean SUV obtained by SC PET/CT between L type and M type, or LL type and L type. The relationship between RC_max_ or RC_peak_ and sphere diameter is shown in Fig. 5. Both of the RC_max_ and RC_peak_ of L type were the highest, but the differences were not statistically significant. The RC_max_ was affected by statistical noise for both PET/CT, while the RC_peak_ was stable and robust for statistical noise. However, RC peak is susceptible to underestimation of quantitative value due to partial volume effect.

## Discussion

The purpose of this study was to investigate whether the image quality and quantification accuracy were affected by the patient’s body mass on ^89^Zr PET/CT. Expectedly, we found that 10-mm-diameter hot sphere could be detected for 5-min acquisition by both SC PET/CT and TOF-PET/CT. However, the detectability depended on PET/CT machines to a similar degree in all types of phantoms.

The N_10mm_ reflects background variability, and all the N_10mm_ examined in our study is greater than the proposed reference level. The technique of PSF and TOF contribute to improving contrast in the 10-mm-diameter hot sphere and resulted in increased background variability. The Q_H,10 mm_/N_10mm_ assures 10-mm-diameter hot sphere visibility, but this metric depends on type of PET/CT scanner models and acquisition duration. In our study, the SC PET/CT showed enough Q_H,10 mm_/N_10mm_ greater than the proposed reference level except for LL phantom with 5-min acquisition. However, the Q_H,10 mm_/N_10mm_ of TOF PET/CT was mostly less than the proposed reference level at 5- or 10-min acquisition in all phantoms. The Q_H,10 mm_/N_10mm_ implies information of the 10-mm-diameter hot sphere contrast and the background variability, and the balance of contrast and noise is valuable for visual detectability of small hot lesion. In this context, long scan duration would be required for TOF PET/CT. All the CV_BG_ calculated in our study were greater than the proposed reference level and decreased with acquisition duration for both PET/CT. The CV_BG_ is reproducible metric and has been used for standardization of ^18^FDG PET/CT in oncology [16–20]. The use of CV_BG_ might facilitate international standardization to reduce variability and global ^89^Zr PET/CT studies [21]. However, the CV_BG_ cannot reflect the effective spatial resolution of the scanner as directly as the RC_max_ or RC_peak_. Both metrics are recommended to standardization on ^89^Zr PET/CT.

In our study, we found that TOF PET/CT was capable of enough detectability by 10-min acquisition per bed position when scanning patient of middle-sized body mass. However, when we scan patient of large-sized body mass, at least 15-min acquisition per bed position would be preferable. In contrast, our findings that 5-min acquisition per bed position by SC PET/CT represented enough detectability when scanning patient of middle-sized body mass. It should be highlighted that SC PET/CT machine is adequate for whole-body scan of ^89^Zr PET/CT. Nevertheless, when we scan patient of large-sized body mass, at least 10-min acquisition per bed position is required.

Our study showed that the image quality and quantification accuracy was affected by the patient’s body mass on ^89^Zr PET/CT. These results were mirrored to the previous results of ^18^F-FDG PET/CT. The main differences between ^18^F and ^89^Zr are the practical range of positron provided by ^18^F and ^89^Zr and cascade γ-ray (909 keV) from each isotope. How these two issue affect image quality was not fully elucidated, but our results revealed that affection to detectability and accuracy were clinically limited.

Both of the RC_max_ and RC_peak_ of L type were the highest among all phantoms, but the differences were not statistically significant. The precise reason why the L type showed the highest values was unclear, but it may be due to large contribution of noise specific to the L type, or inaccurate alignment of the 10-mm hot sphere to the slice center. These can be verified by further multiple re-examinations.

Kaalep and the colleagues suggested that RC curves derived from ^89^Zr phantom using quantitative metrics of RC_max_ and RC_peak_ resulted in increased variability possibly due to activity measurement and phantom filling procedures [12]. This observation is in agreement with our results, because when we investigated both of SC PET/CT and TOF PET/CT, we observed that there was variable bias in the relationship between RC_max_ or RC_peak_ and sphere diameter. Moreover, we observed that RC_peak_ showed variable bias minimally was similar to the results of the previous study using calibration QC and NEMA phantom QC (12). Altogether, the observation discussed above indicate that RC_peak_ is suggestive metric for data comparison among PET/CT systems.

## Conclusions

We demonstrated effects of patient body mass on image quality and quantification accuracy of images obtained using ^89^Zr PET/CT, indicating that acquisition time should be changed according to the patient’s body mass. The RC_peak_ shows minimal variability compared to RC_max_ on ^89^Zr PET/CT, but the underlying precise mechanism of this evidence is unknown. Further investigation is required to clarify optimal metrics for comparison among PET/CT systems.

## Data Availability

Not applicable.
